# Atlantic City, 1901

**Published:** 1901-09

**Authors:** 


### ATLANTIC CITY, 1901.

THE selection of the Hotel Rudolf as the headquarters of the convention fixes the stay of the delegates and their friends in one of the most beautifully located hotels on the island. Its facilities for handling its guests afford everything that could be desired. On the ocean front, it is sure to contribute much to the comfort that so often has been missing at convention sessions in the large cities, where the torrid weather, often experienced early in September, made hours of sitting irksome and distressing. At the upper end of the boardwalk there will be less number of attractions to divert the attention of those in attendance or disturb the deliberations of the body. When the sessions are over there is every attraction of rest, music, social features, an elegant café, and lounging rooms within the hotel, and the bathing beach directly in front, to please and gratify the most fastidious.

---

Governor Voorhees, of New Jersey, is the first Governor to recognize the value of veterinarians in the Good Roads Commission. The precedent thus established we have no doubt will be followed by others when Governors consider the scope of practical knowledge and experience that well-trained veterinarians possess.

